# Ruthenium dioxide nanoparticles as a high-capacity transducer in solid-contact polymer membrane-based pH-selective electrodes

**DOI:** 10.1007/s00604-019-3830-x

**Published:** 2019-11-15

**Authors:** Nikola Lenar, Beata Paczosa-Bator, Robert Piech

**Affiliations:** Faculty of Materials Science and Ceramics, AGH University of Science and Technology, Mickiewicza 30, PL-30059 Krakow, Poland

**Keywords:** Ruthenium dioxide, Nanoparticles, High-capacity transducer, Ion-to-electron transducer, All-solid-state, Solid-contact, Ion-selective electrode, pH potentiometric sensor, Stable potential

## Abstract

A new approach is presented for the design of ion selective electrodes. Ruthenium dioxide nanoparticles were incorporated into solid-contact electrodes, and their properties were studied for the case of pH-selective electrodes. The use of the RuO_2_ is shown to significantly improve the potentiometric response, while no redox response is observed. The use of RuO_2_ results in a Nernstian slope (59 mV/decade) towards hydrogen ions over a wide linear range (pH 2 to 12). The results obtained by chronopotentiometry reveal small resistance, and the capacitance is as high as 1.12 mF. This results in a good stability of the response and in a low potential drift (0.89 μV∙s^−1^). The electrodes exhibit properties nearly as excellent as those of a glass electrode, but they are much smaller, less fragile, and easy to use.

**Graphical abstract:**
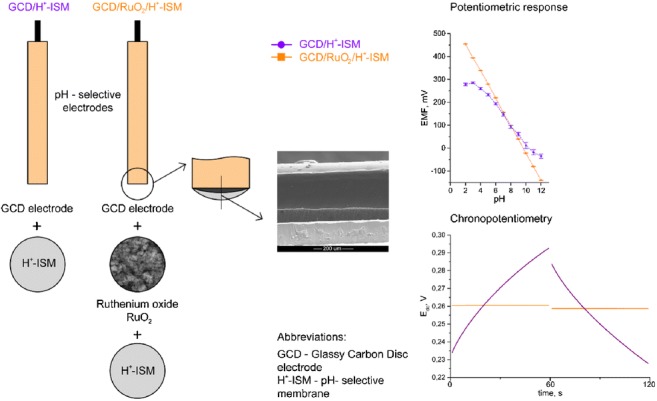
Schematic representation of the construction of the new kind of electrodes along with calibration and chronopotentiometric plots compared to non-modified GCD/H^+^-ISM and modified GCD/RuO_2_/H^+^-ISM electrodes, respectively. The use of ruthenium dioxide results in a wide analytical pH range (2–12) and in high electrical capacitance (1.12 mF).

Schematic representation of the construction of the new kind of electrodes along with calibration and chronopotentiometric plots compared to non-modified GCD/H^+^-ISM and modified GCD/RuO_2_/H^+^-ISM electrodes, respectively. The use of ruthenium dioxide results in a wide analytical pH range (2–12) and in high electrical capacitance (1.12 mF).

According to IUPAC and other sources [1, 2, 3] potentiometry is the only accurate method of pH measurement. Measurements of pH are normally performed using the glass electrode due to its high selectivity and wide pH measurement range [4]. However, its construction results in number of limitations and the main one is its fragility what makes the electrode vulnerable to any mechanical damage and large size, which preclude the analysis of small volume samples [5]. Conventional electrodes such as glass electrode are associated with disadvantages caused by the presence of an inner solution. This construction of an electrode requires vertical position, stable temperature and pressure to avoid the phase change of the inner solution [6]. To overcome those shortcomings, the inner solution must have been removed from electrode construction. The first construction of a solid-contact electrode was, so called, coated-wire electrode of a simple construction but poor potential stability and irreproducibility caused by the low double layer capacitance and high resistance of the charge transfer between the ion-selective membrane (ISM) and the electronic conductor [7]. The transduction between the electronic conductor and the ionic conductor (ion – selective membrane) became possible when the internal layer which exhibits both types of transduction (electronic and ionic) was implemented into the electrode construction [8].

Various electroactive materials such as conducting polymers [9] and organic conducting crystals [10] which exhibit high redox capacitance, as well as nanomaterials transducers [11] (such as microfibers, microspheres, nanoparticles and microcapsules), nanoporous gold [12] and carbon black [13, 14] characterized by a high double layer capacitance have been already used as internal layers. Metal oxides also appeared to improve electrodes’ properties. Zeng and Qin [15] used MoO_2_ as solid-contact layer and observed the impact on electrical parameters, resulting in stable and rapid potential response.

In this paper the new approach was presented by the use of ruthenium oxide nanoparticles as a material for high-capacity transducer to construct a solid contact H^+^-selective ISE with a PCV membrane. To the best of our knowledge this material has never been used as solid-contact layer in ion selective electrodes’ construction.

Ruthenium dioxide (RuO_2_) characterized as transition metal dioxide with a rutile structure is of both scientific and technological importance, what results from its unique properties. Ruthenium dioxide exhibits a combination of unique characteristics such as high thermal and chemical stability, low resistivity, and remarkable redox properties [16] what was taken advantage of when designing the ISE.

We are presenting here a universal approach which will be implemented into further studies over ISEs including improving parameters of electrodes and expanding the analytes range. To the best of our knowledge this is the first article concerning this kind of application of ruthenium oxide as solid contact layer in all-solid-state electrodes.

## Experimental

### Chemicals

Membrane components: hydrogen ionophore V (Calix[4]-aza-crown), sodium tetrakis(4-fluorophenyl)borate dihydrate, 2-Nitrophenyl octyl ether (NPOE) and high molecular weight poly(vinyl chloride) were purchased from Sigma-Aldrich (www.sigmaaldrich.com). Dimethylformamide (DMF) and Tetrahydrofuran (THF) used as solvents were also purchased from Sigma-Aldrich. Ruthenium dioxide (RuO_2_) used as solid-contact layer was purchased from Acros Organics (www.acros.com). Other chemicals (including TRIS, citric acid, boric acid, sodium hydroxide, potassium hydroxide, lithium hydroxide and sodium chloride) were used for further analysis in form of aqueous solutions. Aqueous solutions were prepared by dissolving salts and acids in distilled and deionized water and then titrated with sodium hydroxide and hydrochloric acid to meet the desired pH values. All chemicals were of analytical grade and were used as received without any further purification.

### Electrodes preparation

To determine the impact of RuO_2_ solid contact layer on the all-solid-state pH-selective electrodes’ properties solid-contact electrodes and a coated-disc electrode were developed. The solid contact layers were obtained by dispersion of ruthenium oxide (5 mg) in DMF (1 mL) and oxide’s implementation onto Glassy Carbon Disc (GCD) electrodes using drop casting method. Before casting, the electrodes were cleaned: first by polishing them with alumina slurries (particle size of 0.3 μm and 0.05 μm), then rinsed with water and finally cleaned ultrasonically with water and methanol. Cleaned and dried GCD electrodes were then covered by dropping 20 μl of ruthenium oxide DMF solution in order to prepare RuO_2_-modified electrodes. Mediation layers were dried in elevated temperature for several minutes. The hydrogen selective membrane cocktail was obtained by dissolution 252 mg of the membrane components in 2 mL of THF. The composition of H^+^- ISM was as follows: 0.90% (*w*/w) hydrogen ionophore V, 66% (w/w) o-NPOE, 32.85% (w/w) PVC, and 0.25% (w/w) sodium tetrakis(4-fluorophenyl)borate dihydrate. Then, solid contact layers were covered twice with 30 μL of ion-selective membrane solution in order to obtain solid-contact H^+^-selective electrodes (GCD/RuO_2_/H^+^-ISM). Additionally, the coated disc electrodes (GCD/H^+^-ISM) were prepared as controls by covering glassy carbon disc electrodes with above mentioned membrane.

Afterwards, all electrodes were left to complete solvent evaporation at room temperature for 24 h. Dried electrodes were conditioned in 0.1 M NaCl solution for 1 day and then the conditioning procedure was repeated prior to every measurement. Three identical electrodes were prepared and all of them were studied.

### Electrochemical and electron microscopic measurements

Potentiometric measurements were performed using a 16-channel mV-meter (Lawson Labs, Inc., Malvern, PA) (www.lawsonlabs.com). The reference electrode was an Ag/AgCl electrode with 3 M KCl solution (6.0733.100 Metrohm, Switzerland) (www.metrohm.com) The potentiometric response towards H^+^ was measured by recording calibration plots for the electrodes in standard solutions buffered with 10 mM citric acid and 10 mM boric acid titrated with sodium hydroxide or hydrochloric acid. The pH range of buffer was 2 to 12. The selectivity measurements were carried out in Tris buffered solutions with the concentration of studied cations of 10^−1^ M using Fixed Interference Method. Potentiometric measurements were conducted in the presence of glass electrode (6.0150.100 Metrohm, Switzerland).

The chronopotentiometry and electrical impedance spectroscopy measurements were conducted with the use of an Autolab General Purpose Electrochemical System (AUT302N.FRA2-AUTOLAB, Metrohm Autolab, The Netherlands) (www.metrohm-autolab.com). Studied ion-selective electrodes were being connected in sequence as working electrodes into three-electrode cell with an Ag/AgCl/3MKCl electrode as the reference electrode and a glassy carbon rod as the auxiliary one. All *data* analysis *was* carried out and interpreted using *NOVA 2.1.*

Chronopotentiometric studies were carried out in buffer of pH 3. A constant current of +1 nA was applied to the working electrode for 60 s, followed by a − 1 nA current for another 60 s.

The electrochemical impendence spectroscopy (EIS) measurements were performed in buffer of pH 3. Impedance spectra were measured by applying a frequency from 100 kHz to 0.01 Hz using an AC amplitude of 50 mV superimposed on open-circuit potential (OCP).

The morphologies of the ruthenium oxide as well as ion-selective membrane were examined with the use of a scanning electron microscope, SEM model LEO 1530 from LEO Electron Microscopy, Carl Zeiss¸ Germany (www.zeiss.com).

## Results and discussion

### Choice of material

Ru-based materials are well-known supercapacitors (electric double layer capacitors), which electrical performance is strongly connected to the surface area [17].Because of its unique assets such as high chemical stability, low resistivity, and remarkable redox properties [16], ruthenium dioxide is an excellent material for preparing electrodes [18, 19].In our ion-selective all-solid-state electrodes, ruthenium dioxide nanoparticles were used to create a solid-contact layer placed between ion-selective membrane and electronic conductor.One of the approaches to improve properties of all-solid-state ion-selective electrodes is to implement the material characterized by high electrical capacitance. This can be done in two ways: either by using the material of high surface area or the material which displays high redox capacitance.

Ruthenium dioxide turned out to exhibit both mentioned properties. Thanks to the nanometric size of its particles (revealed by Scanning Electron Microscopy), oxide is characterized by the high surface area and this was the first reason to consider ruthenium dioxide as promising material to be applied as solid-contact layer in ion-selective electrodes. Besides its favourable morphology, ruthenium dioxide act as an “ion-to-electron” transducer. One of the mechanisms proposed by Fog and Buck in [20] is that in contact with hydrogen ions oxide undergoes redox reaction in which ions and protons are being exchanged. This mechanism decides of oxide’s ability to transduce both ions and electrons through the layer, which simplifies the processes on the interface between ion-selective membrane and electronic conductor.The combination of those two properties: high surface area and high redox capacitance [6] in one material, used as solid-contact layer allowed to obtain electrodes characterized by stable potentiometric response.

One other feature that needs to be taken into consideration when designing the robust ion-selective electrodes is the wettability of chosen material. This is of particular importance in terms of creating the water film between membrane and electronic conductor which may lead to deteriorating membrane adherence [21]. Hydrophilic materials tend to create the water layer, making the electrode prone to damages, whereas hydrophobic materials are said to prevent the occurrence of water layer. In our studies, despite of ruthenium dioxide’s hydrophilic properties (as we presented in [22]) and extensive conditioning (time of conditioning equal to 3 months), during the water layer test we did not observed characteristic features indicating the presence of water film (not shown here). This may be caused by the specific reaction that occurs on the surface of RuO_2_-layer [20] or/and the high surface area that determines strong adherence between solid-contact layer and membrane, preventing its detaching from electrode’s surface.

The choice of ruthenium dioxide for solid-contact layer in pH-selective electrodes turned out to be appropriate and allowed to obtain pH-sensor which exhibit remarkable analytical and performance parameters described in the paper.

### Scanning electron microscopy

**Fig. 1:**
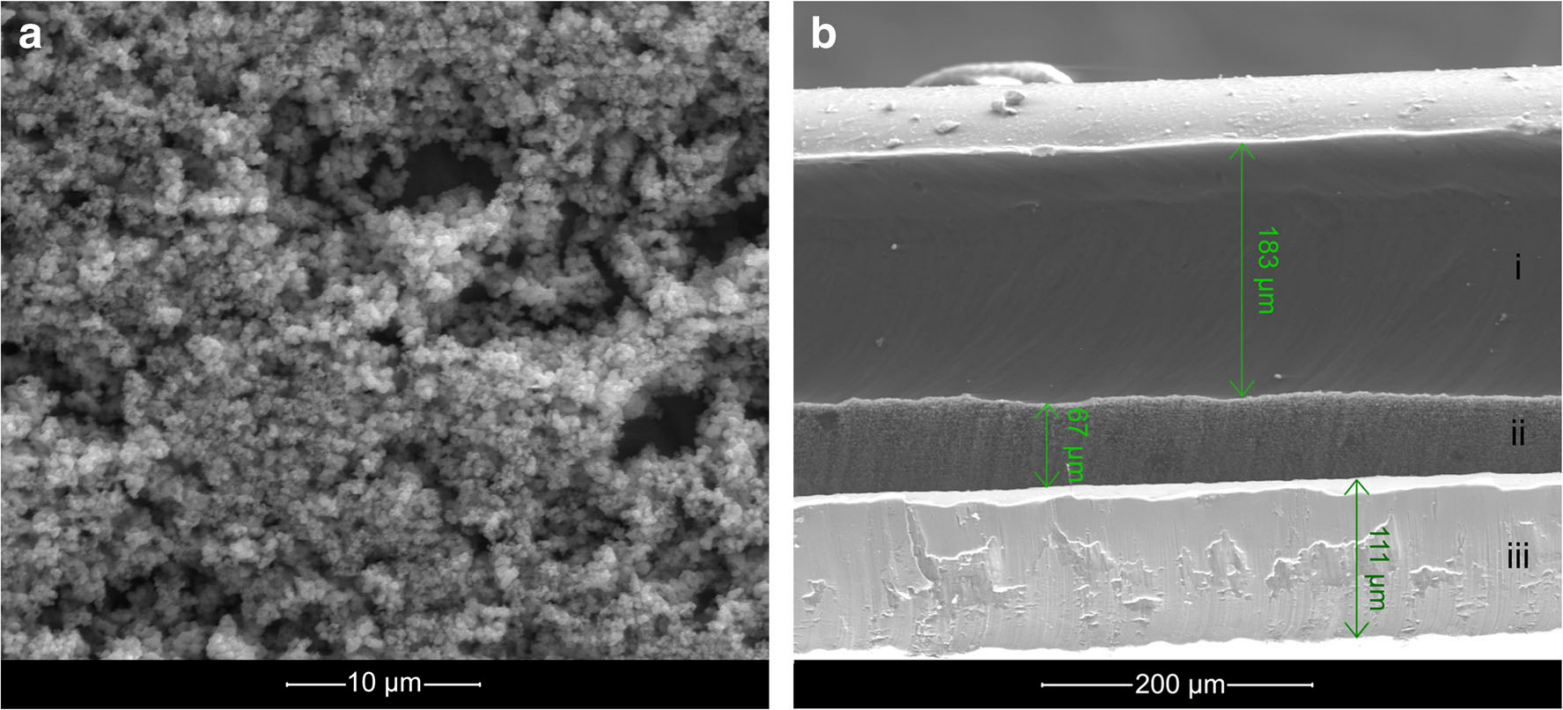
SEM images showing the morphology of **a** ruthenium oxide, **b** profile of ion-selective membrane (i) and ruthenium oxide (ii) as internal layer casted onto titanium pad (iii) with the estimated thickness of the layers

The SEM images showing the morphology of the ruthenium oxide nanoparticles RuO_2_ used as the intermediate layer are presented on the Fig. 1. Figure 1a shows the oxide’s morphology which was casted onto titanium pad of 1cm^2^ surface. Figure 1b presents the profile of layers: ion-selective membrane (i), ruthenium oxide (ii) and titanium pad (iii) along with thickness of layers after casting the pad with 20 μl of RuO_2_ and 60 μl of membrane.

### Potentiometry measurements

**Fig. 2:**
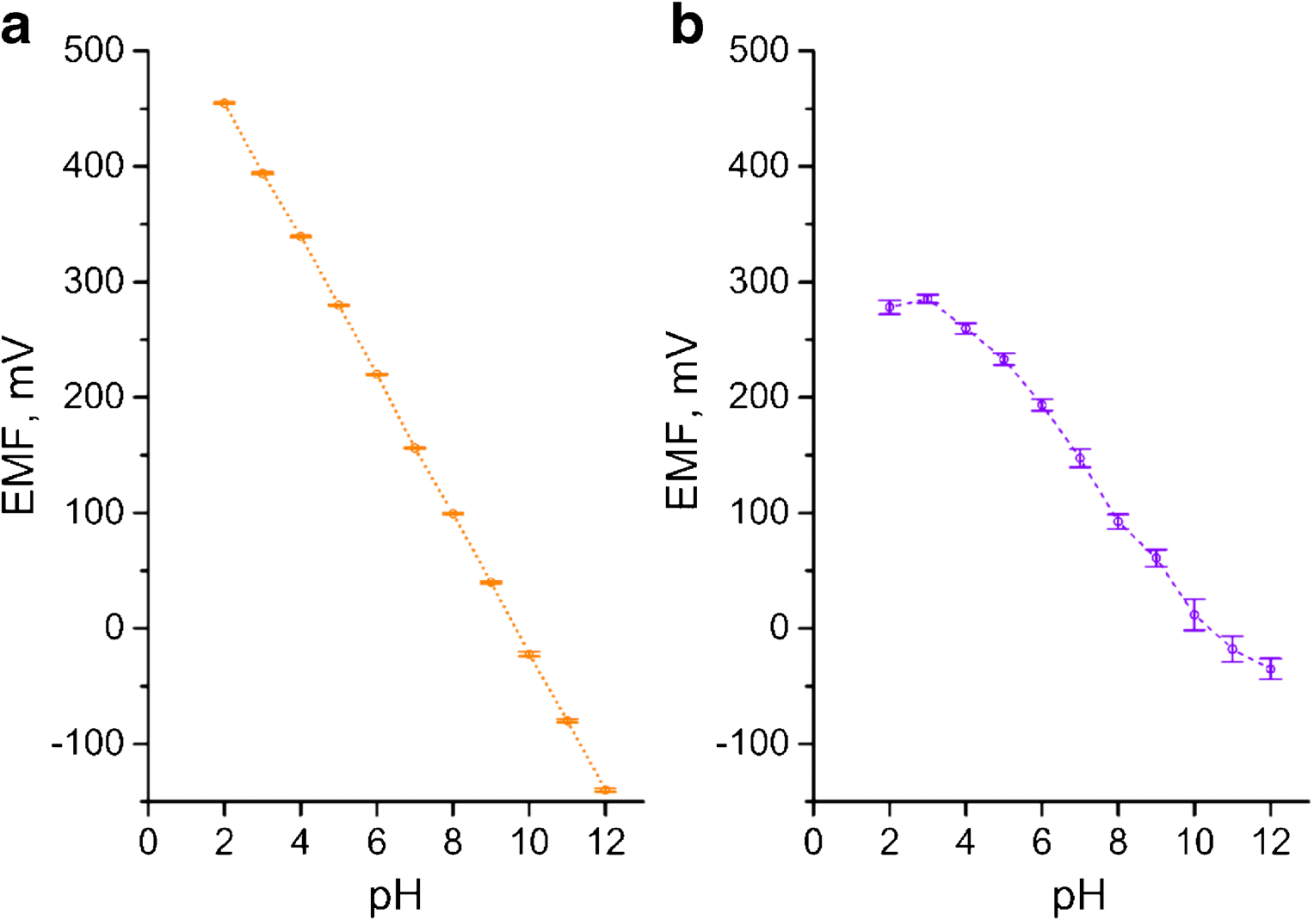
Mean EMF values with SD recorded over 6 calibrations of (**a**) GCD/RuO_2_/H^+^- ISM electrode and (**b**) GCD/H^+^- ISM electrode performed over one week (*n* = 6 calibrations)

The potential responses of the studied solid-contact electrodes (GCD/RuO_2_/H^+^-ISM) were measured in aqueous critic and boric acid buffer of pH range 2–12. The pH was adjusted to desired value with NaOH and HCl used as a titrant. The calibration plot slope of examined electrodes were compared with coated disc electrodes with ion-selective membrane attached directly to glassy carbon electrode’s surface (GCD/H^+^-ISM). Table 1 presents the average values of slope and standard potential obtained from 6 calibration plots recorded during one week of conditioning in 0.1 M NaCl (after 24, 48, 72, 96, 120 and 148 h) for one electrode of each kind. The slope and standard potential values were determined for the linear range of calibration plot. Figure 2 shows the exemplary dependency of the recorded electrodes potentials on the pH for modified all-solid-state electrode after conditioning in 0.1 M NaCl solution. All results obtained for studied electrodes are summarized in Table 1.

**Table 1:** Comparison of repeatability of coated wire and RuO_2_-modified electrodes (based on 6 calibrations conducted over one week *n* = 6)

Electrode	Slope S ± SD [mV/pH]	Standard potential E^0^ [mV]	Linear range [M]
GCD/RuO_2_/H^+^-ISM	59.31 ± 0.15	571 ± 2	10^−2^ - 10^−12^
GCD/H^+^-ISM	46.86 ± 4.60	550 ± 14	10^−4^ - 10^−10^

The reproducibility of electrodes was evaluated after 48 h of conditioning based on results obtained for 3 identical electrodes prepared at the same time, using identical materials and technique. Averaged values of standard potential and calibration plot slope together with standard deviation values are presented in the Table 2.

**Table 2:** Comparison of reproducibility of coated wire and RuO_2_-modified electrodes (*n* = 3, conditioning time equal to 48 h)

Electrode	Slope S ± SD [mV/pH]	Standard potential E^0^ [mV]	Linear range [M]
GCD/RuO_2_/H^+^-ISM	59.05 ± 0.18	571 ± 1.7	10^−2^ - 10^−12^
GCD/H^+^-ISM	48.72 ± 3.3	556 ± 12	10^−4^ - 10^−10^

The GCD/RuO_2_/H^+^- ISM electrode showed Nernstian response in wide range of pH value (2–12) and the slope value was stable and close to theoretical value with the increasing time of conditioning. The GCD/RuO_2_/H^+^-ISM electrode exhibited great repeatability and reproducibility (given by standard deviation values) as it is shown in the Table 1 and Table 2, respectively. For the coated-wire electrode the narrow lineal range of pH was obtained, and the near-Nernstian response was observed from pH = 5 to pH = 10 only after 24 h of conditioning. In further calibrations GCD/H^+^-ISM electrodes exhibited worse performance.

**Fig. 3:**
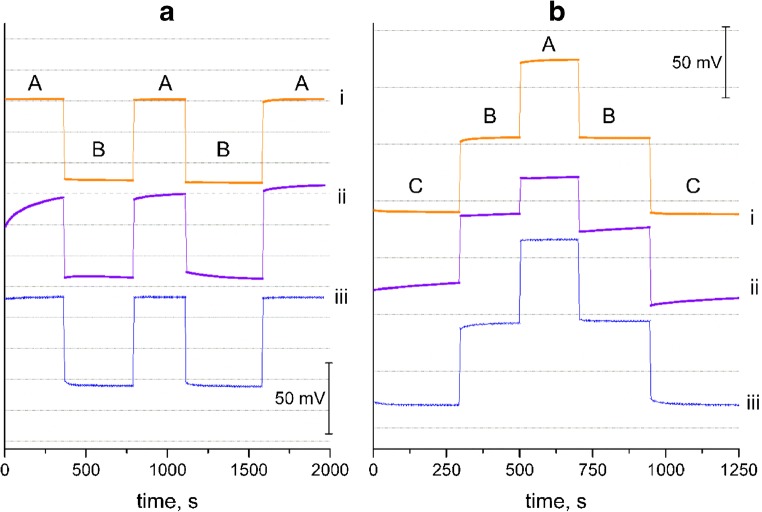
Electrodes’ response reproducibility tested by measuring EMF in **a** pH = 4, **b** pH = 5 and **c** pH = 6 buffer (i – GCD/RuO_2_/H^+^- ISM, ii - GCD/H^+^- ISM, iii – glass electrode)

Afterwards the potentiometry response was tested in buffer of pH 4, 5 and 6 in order to observe the stabilization of electrode’s response after the change of pH value and to test their reversibility. Figure 3 shows reversibility test made for GCD/RuO_2_/H^+^- ISM, GCD/H^+^- ISM (after 24 h of conditioning in 0.1 M NaCl) and glass electrode.

Fig. 3 shows that the RuO_2_-contacted electrode exhibited almost as good stabilization as glass electrode in contrast to coated-wire electrode.

The measurements confirmed a good reproducibility of GCD/RuO_2_/H^+^- ISM electrode, for which registered potential was repetitive when measuring the solution of particular pH value. RuO_2_ - contacted electrode showed highly reversible potential response towards hydrogen ions.

### Redox test

**Fig. 4:**
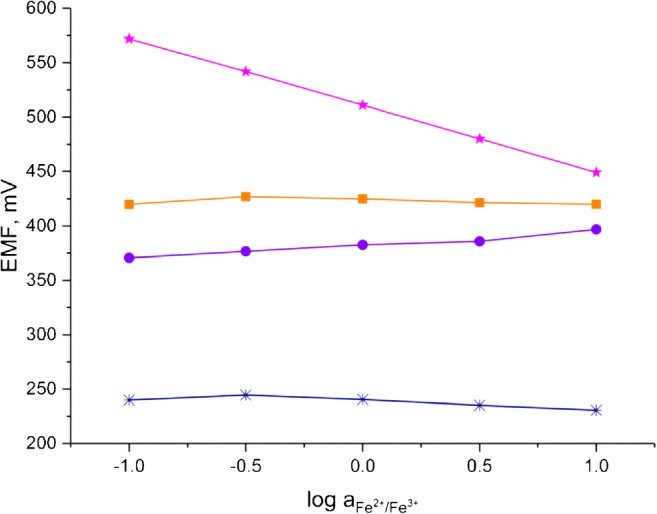
Redox response compared for glass electrode (), RuO_2_ – modified electrode (), coated-wire electrode () and platinum electrode ()

The redox sensitivity measurements were conducted for the solid contact electrodes (GCD/RuO_2_/H^+^-ISM) as well as for the coated-wire electrodes (GCD/H^+^-ISM) with the glass and platinum electrode used as a control one. The potential stability was recorded in solutions containing constant amount of a FeCl_2_ and FeCl_3_ redox couple (1 mM) with the log of Fe^2+^/Fe^3+^ ratio equal to −1, −0.5, 0, 0.5 and 1. The results obtained are presented in the Fig. 4.

There was no redox response detected in RuO_2_-modified electrodes and a slight change of potential was detected in non-modified ones. The change of potential was caused rather by various pH values in examined solutions than by redox signal. Platinum electrode exhibited near- Nernstian response with the change of log Fe^2+^/Fe^3+^ value (61.50 mV/dec) as expected.

Although solid contact layer of RuO_2_ is an electronic conductor and should exhibit clear redox response, the polymer-based ion-selective membrane is an electronic insulator and eliminates the redox response. Therefore, the examined sensors are not redox sensitive.

### Selectivity coefficients

The information about the selectivity of ion-selective electrodes is crucial to determine whether an electrode cannot be used in certain conditions. The value of the selectivity coefficient informs about electrode’s ability to distinguish the primary ion in the presence of interfering ions in the examined solution. Electrode selectivity is determined by the membrane components – mainly ionophore which is responsible for selective recognition and binding of the determined ion. What is important, hydrogen ionophore V used for preparing GCD/RuO_2_/H^+^-ISM electrode shows selectivity for divalent ions such as Mg^2+^ or Ca^2+^ due to the presence of ﻿t-butyl groups in its structure [23]. No influence of these ions on the electrode potential was observed even with their large excess compared to hydrogen ions.

**Fig. 5:**
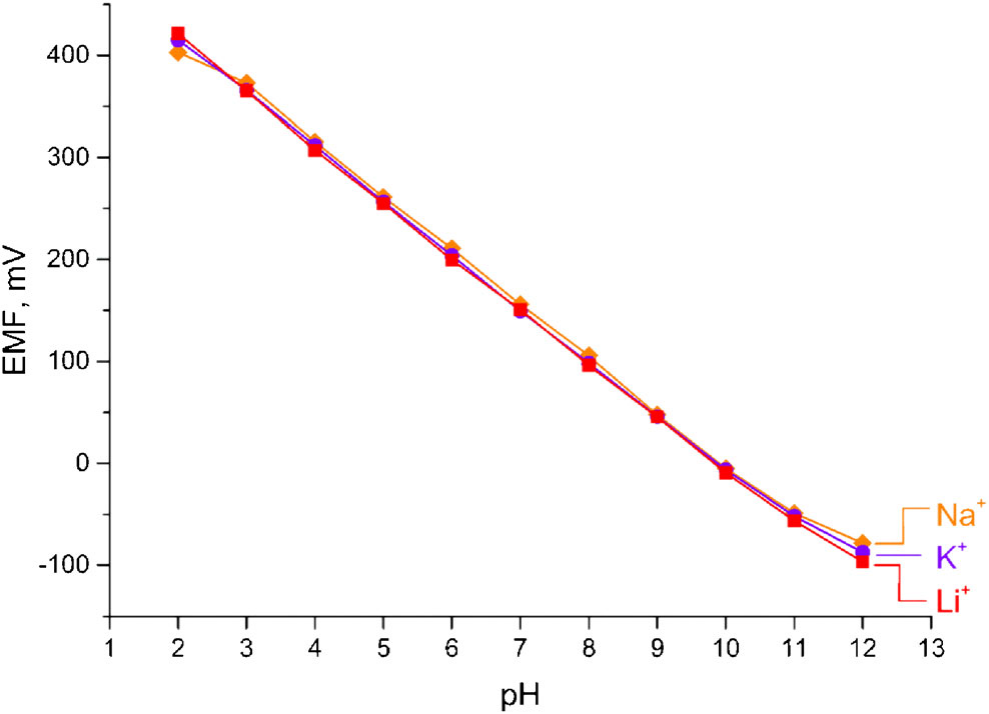
Exemplary potentiometric response of GCD/RuO_2_/H^+^-ISM electrode measured in TRIS-HCl buffer of pH range 2–12 containing 0.1 M of NaOH, KOH and LiOH

However, as shown in the literature, the working range of electrodes based on macrocyclic compounds can be affected by the presence of monovalent cations such as lithium, potassium and sodium at high pH [24]. For this reason it was checked whether the introduction of ruthenium oxide layer does not change the electrode selectivity towards monovalent ions. The potentiometric selectivity of the studied H^+^-selective electrodes for interfering ions (Na^+^, K^+^ and Li^+^) was evaluated with the Fixed Interference Method (FIM). The FIM (Fixed Interference Method) involves measuring electromotive force (EMF) in a solution with constant interfering ion activity and increasing primary ion activity. The determination of selectivity coefficient is based on the graph of EMF in function of logarithm of primary ions’ activity (Fig. 5).

Interfering cations were examined in form of the hydroxides (NaOH, KOH, LiOH) and their concentration in the analysed solutions was of 10^−1^ M. Prior to the measurement the ISEs were conditioned in pH = 3 buffered solution for 1 h. To determine the selectivity of studied electrodes, their responses were recorded in buffered TRIS solutions of constant hydroxide concentration, titrated with hydrochloric acid, while the pH was monitored with the glass electrode to achieve solutions of pH range 2 to 12. The selectivity sequence for the studied electrodes was: Li > K > Na. Obtained results were consistent with the results presented in [23] and there was no potentiometric response towards interfering cations observed from pH = 2 to pH = 12.

In summary, the addition of ruthenium oxide as solid contact does not affect the selectivity of investigated H^+^-selective electrodes, as expected. Similar results were observed when using carbon nanomaterials [13, 25, 26].

### Potential stability

**Fig. 6:**
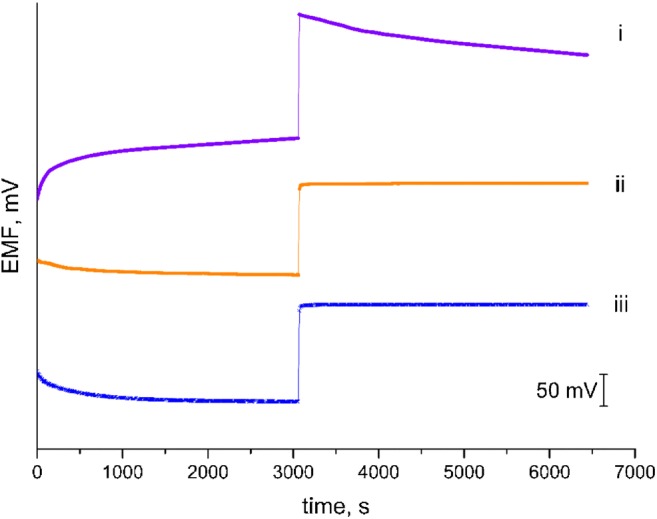
EMF stabilization in 10^−3^ M solutions of KCl and HCl compared for coated-wire (i), solid-contact (ii) and glass (iii) electrodes

The stability of electrodes’ response with time was tested using 10^−3^ M KCl and 10^−3^ M HCl. RuO_2_-modified electrodes were compared with control electrode and their response in both solutions was measured. Figure 6 presents the stabilization of potential with time while changing the solution from 10^−3^ M KCl to 10^−3^ M HCl and starts immediately after the solution exchange from 10^−3^ M HCl to 10^−3^ M KCl. The GCD/RuO_2_/H^+^-ISM electrode exhibits remarkable properties comparing to coated-wire electrode. It can be seen that a shorter time is needed for solid-contact electrode to reach a stable potential than for the coated-wire one.

Afterwards the electrodes were left for 15 h in 10^−3^ M HCl solution to determine their potential stability over the time.

**Fig. 7:**
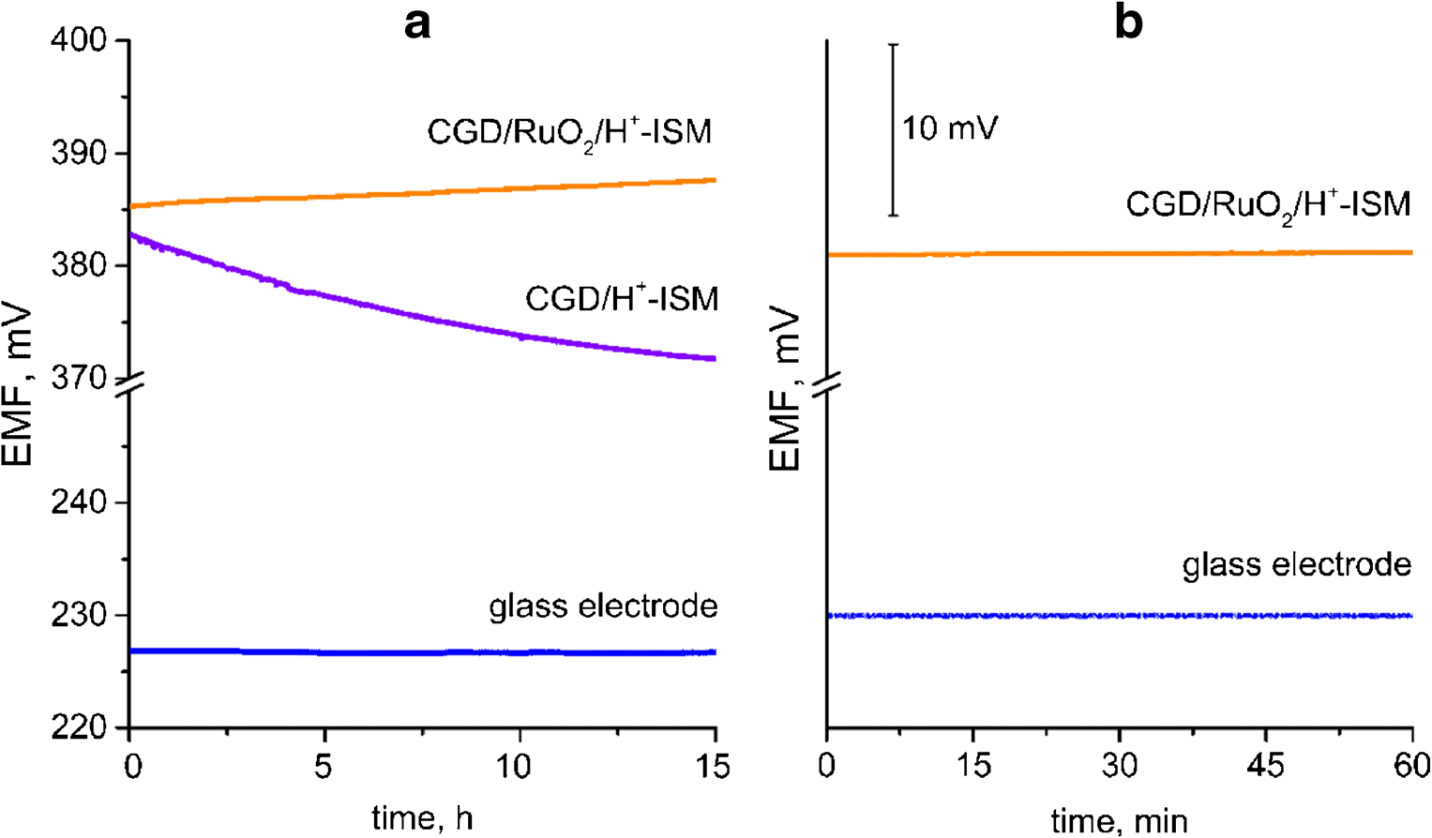
Stability of electrodes’ response over: **a** 15 h, **b** 1 h measured in 10^−3^ HCl solution

For the solid-contact electrode the stability given by ratio dE/dt was better (0.15 mV∙h^−1^) than for coated-wire electrode (dE/dt = 0.73 mV∙h^−1^) what can be seen on Fig. 7.

Studied electrodes have been tested over 3 months and during this period of time all of them displayed stable potentiometric response. After 3 months of intensive tests carried on electrodes, the membrane adherence was not deteriorated, electrodes were not damaged and the potential response was stable, therefore the lifetime of studied GCD/RuO_2_/H^+^-ISM electrodes can be estimated at over 3 months.

Analytical parameters obtained for studied GCD/RuO_2_/H^+^-ISM electrode were compared with parameters presented in previous articles on pH-selective ISEs, micro electrodes and nano electrodes. The compilation is presented in the Table 3.

**Table 3:** An overview on recently reported potentiometric methods for determination of hydrogen ions with solid contact electrodes

Electrode/ Solid contact material used for electrode construction	Linear range [M]	Slope [mV/pH]	Standard potential E^0^ [mV]	Time of response [s]	Ref.
Polypyrrole doped with hexacyanoferrate(II) (PPy-Fe(CN))	2–12	56.9 ± 4.3	519 ± 21	10	[27]
Multi-walled carbon nanotube (MWCNT)	2.89–9.90	58.8 ± 0.4	–	<10	[5]
Polydopamine - carbon nano-onion (CNO – PDA)	1.50–10.50	60.1 ± 0.3	–	–	[28]
Polyaniline (PANI)	2–9	52.7 ± 1.1	–	60–120	[29]
Nano Electrode: Polyaniline deposited onto a carbon fiber (PHNE)	2.0–12.5	60*.*0 ± 0*.*5	–	6 s (pH 7)	[30]
Nano Electrode: carbon nanotube deposited onto polyaniline (CFCNEs)	1–13	58	–	few seconds	[31]
Polypyrrole (PPy) doped with cobaltabis(dicarbollide) ions ([3,3′-Co(1,2-C2B9H11)2]−)	3.5–11	52 ± 2	–	<45	[32]
Poly(3,4-ethylenedioxythiophene) − poly(styrenesulfonate) (PEDOT(PSS))	3–11	57.7 ± 0.2	–	–	[33]
Ruthenium dioxide (RuO_2_)	2–12	59.31 ± 0.15	571 ± 2	5	this work

As presented in the table, electrodes with ruthenium dioxide as solid-contact layer are characterized by the linear range complementary to the previous solutions described in literature. Applying ruthenium dioxide allowed however to receive the calibration plot with slope value equal to 59.31 mV/pH which is in very good agreement with the theoretical Nernstian value. Obtained electrode can be also described with the outstanding repeatability represented with the standard deviation values from averaged values of slope and standard potential.

Time of response (t_0.95_) calculated for RuO_2_-contacted ISEs following the IUPAC principles equals 5 s. Amongst compared potentiometric methods, the shortest response time can be attributed to nano-electrodes for which the time needed to reach the stable potential equals merely few seconds. In comparison with presented nano-electrodes, studied ISEs displayed comparable time of response.

Despite being characterized by similar analytical parameters, the main difference between nano-sized electrodes and studied ISEs is that the size of nano-sensors can be narrowed to 100 nm, whereas glassy carbon electrodes are at least few centimetres long.

What should be emphasized here is that solid-contact materials presented in the Table 3, which exhibit satisfying analytical parameters such as wide linear range (which allow to measure at least 9 pH values) are the material composites (consist of two varying materials). Ruthenium dioxide applied alone and without any modifications, allows to obtain the ion-selective electrode of parameters that are comparable to the best potentiometric methods described in literature.

### Chronopotentiometry measurements

**Fig. 8:**
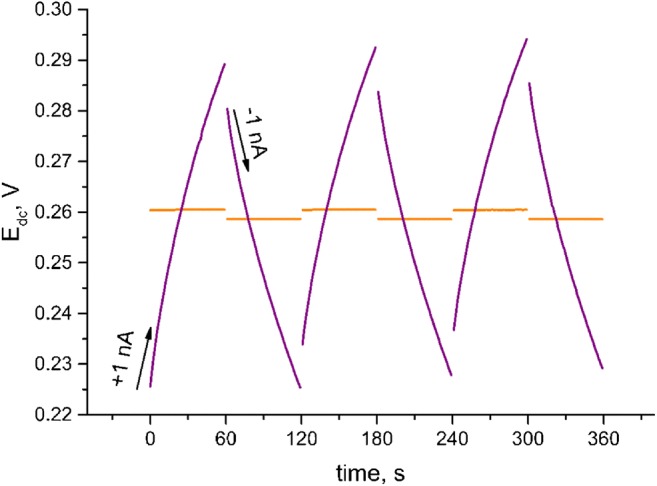
The response of electrodes measured during 1 nA and − 1 nA current flow (orange - GCD/RuO_2_/H^+^-ISM, violet - GCD/H^+^-ISM); inset: the response of GCD/RuO_2_/H^+^-ISM electrode recorded during 120 s of forced current flow

The chronopotentiometry method was used in order to check the influence of the current flow through the measuring cell on the stability of the electrode response signal. In constant current chronopotentiometric experiments, a known, set current (I) is applied to an electrode and the potential is recorded over time. In this case the measurement was carried out with the forced flow of 1 nA current. Based on the registered potentiometric plots, it is possible to estimate such parameters of ion-selective electrodes as resistance, potential drift and electrical capacity. With the ion-selective electrodes, high electrical capacities are desirable for their low resistance and low polarizability. The high electrical capacity also determines the stability of the potential response (low potential drift dE/dt) and the possibility to use the sensor in current flow conditions. The measuring cell consisted of a reference electrode - a silver chloride Ag/AgCl electrode with 3 M KCl, an auxiliary electrode - glassy carbon rod and working electrode – investigated ion-selective electrode. The measurements (one for each electrode) were carried out in a buffer of pH 3. During the measurement, a current of 1 nA was forced through the cell under test, with the current flow changing every 60 s (+1 nA for 60 s, followed by a − 1 nA current for another 60 s). The change of the current flow occurred 5 times and after each change the potential jump was observed. Based on obtained results, the following parameters were determined: resistance, electrical capacity and potential drift. Electrical parameters were calculated as an average value from 6 steps that were recorded during the measurement and presented on Fig. 8. It should be noticed that the RuO_2_-modified electrodes exhibited low potential response during analyzed period of time comparing to coated-wire electrodes which is shown on Fig. 8.

The total resistance of the electrode was calculated using the value of potential jump ΔE_dc_ and current I: R_total_ = ΔE_dc_/2I. The estimated values were as follow: for solid-contact electrode (GCD/RuO_2_/ H^+^-ISM) R_total_ = 0.90 ± 0.02 MΩ, for coated-wire electrode (GCD/H^+^-ISM) R_total_ = 3.20 ± 0.08 MΩ (*n* = 6).

The potential drift of the electrode was calculated and the capacitance C was obtained with the use of ΔE_dc_/Δt = I/C equation. The high electrical capacity determines the stability of the potential response (low potential drift ΔE_dc_/Δt). For RuO_2_-contact electrodes obtained results for capacitance were approximately 1.12 ± 0.04 mF and potential drift 0.89 ± 0.03 μV∙s^−1^ (n = 6) while for coated-wire electrodes, the capacitance value was lower (0.89 ± 0.04 μF) and potential drift given by ΔEdc/Δt ratio was much higher (1.13 ± 0.05 mV∙s^−1^) (n = 6).

It should be mentioned that the GCD/RuO_2_/H^+^-ISM electrode exhibit incomparably higher capacitance than obtained for ISEs with other solid-contact materials described in literature. Table 4 contains capacitance values obtained for selected materials applied in ion-selective electrodes. As presented, CB- [13], TCNQ- [34], GR- [35] PEDOT [9] or PEDOT-CNT [36] - contacted ISEs are characterized by much lower capacitance value.

**Table 4:** Capacitance values compared for GCD/SC/ISM electrodes with various material applied as solid-contact (SC) layers

Electrode symbol	SC material	Capacitance value [μF]	Reference
GCD/CB/ NO_3_^−^ -ISM	Carbon Black	289	[13]
GCD/TCNQ/K^+^-ISM	7,7,8,8-Tetracyanoquinodimethane	132	[34]
GCD/TCNQ/Na^+^-ISM	7,7,8,8-Tetracyanoquinodimethane	154	[34]
GCD/GR/ K^+^-ISM	Graphene	91	[35]
GCD/PEDOT/Ca^2+^-ISM	Poly(3,4-ethylenedioxythiophene)	45	[9]
GCD/PEDOT(CNT)/K^+^-ISM	Poly(3,4-ethylenedioxythiophene) – Carbon Nanotubes	83	[36]
GCD/MoO_2_/K^+^-ISM	Molybdenum Dioxide	86	[15]
GCD/GR-TTF/NO_3_^−^ -ISM	Graphene - Tetrathiafulvalene	1180	[37]
GCD/CIM/K^+^-ISM	﻿Colloid-Imprinted Mesoporous Carbon	1000	[25]
GCD/CB-GR-FP/K^+^-ISM	Carbon Black – Graphene - ﻿Fluorinated acrylic copolymer	1471	[38]
GCD/PC-SMSs/K^+^-ISM	﻿Porous Carbon - Sub-Micrometer Spheres	2803	[26]
GCD/CB-TTFCl/Cl^−^-ISM	Carbon Black – Chloride salt of Tetrathiafulvalene	2800	[39]
GCD/RuO_2_/ H^+^-ISM	Ruthenium dioxide	1120	this work

Also, it should be emphasized that electrodes with molybdenum dioxide [15] showed capacitance value that is several time smaller than for RuO_2_-contacted electrodes described in this paper.

Obtained capacitance value is comparable to values presented in [37] for GR-TTF -contacted ISE, in [25] for CIM -contacted ISE or in [38] for CB-GR-FP -contacted ISE. However, the values published by [26] for PC-SMSs -contacted ISE and [39] for CB-TTFCl - contacted ISE were significantly higher.

### Electrochemical impedance spectroscopy measurements

**Fig. 9:**
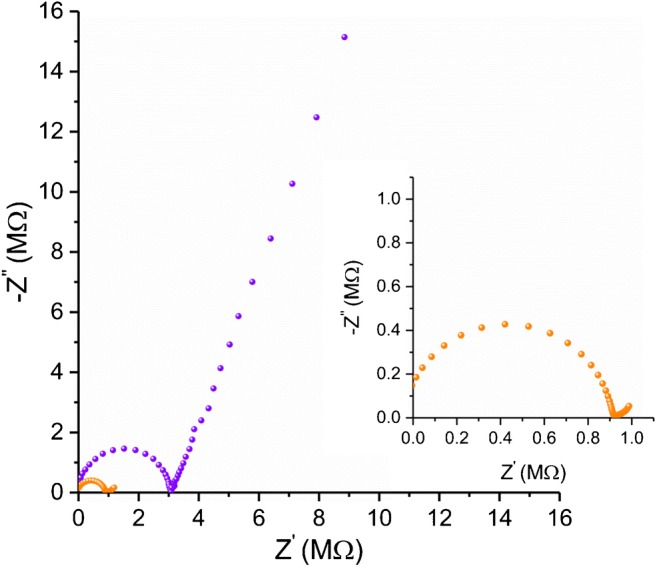
EIS spectra of GCD/RuO_2_/H^+^-ISM () and GCD/H^+^-ISM () recorded in pH = 3 buffer in the frequency range from 100 kHz to 10 mHz, using an amplitude of 50 mV. Inset: scaled graph of GCD/RuO_2_/H^+^-ISM electrode

Representative impedance spectra for ISEs with (GCD/RuO_2_/H^+^-ISM) and without (GCD/H^+^-ISM) RuO_2_ as solid contact are shown in Fig. 9. In the high-frequency region, the diameter of the semicircle represents the bulk membrane resistance (R_b_) together with contact resistance between the ion-selective membrane and underlying electronic conductor. For this reason, after applying the RuO_2_ layer with high surface area, the resistance value decreased about 3.5 times and is equals 3230 kΩ and 954 kΩ for GCD/H^+^-ISM and GCD/RuO_2_/H^+^-ISM, respectively. From the high-frequency region it is possible to obtain also the geometric capacitance value (C_b_), which equals 24 pF and 45 pF for GCD/H^+^-ISM and GCD/RuO_2_/H^+^-ISM, respectively.

The characteristic in the low-frequency region is caused by the double-layer capacitance (C_dl_) coupled with a charge-transfer resistance (R_ct_) at the ISM and underlying electronic conductor interface providing information about the easiness for electron/ion transfer at the electrode interface [40]. The R_ct_ can be separated into two components: the electronic and ionic resistances. The electronic resistance includes the electronic conductivity of the RuO_2_ particles, the electronic contact between particles, and electronic contact between GC material and RuO_2_. The ionic resistance is the membrane ionic resistance inside the pores of the RuO_2_ layer. It depends on the membrane conductivity, porosity of the RuO_2_ layer and its thickness. In the case of very porous layers, as the frequency drops, the AC signal penetrates deeper into the pores and the capacitance increases, which is responsible for the 45° slope seen at high frequencies (seen in the case of GCD/RuO_2_/H^+^-ISM electrode).

The low-frequency part of the EIS for GCD with polymeric H + -selective membrane GCD/H^+^-ISM showed a large semicircle arising from a small capacitance with a large charge-transfer resistance at the blocked GCD│ion-selective PVC membrane interface. The R_ct_ equals 245 MΩ and C_dl_ expressed as *Constant* Phase *Element* CPE_dl_ (Y0) is equal to 0.63 ^(0,8)^ μS·s^(N)^. In the case of GCD/RuO_2_/H^+^-ISM electrode similar value of CPE_dl_ was obtained 0.84 ^(0,7)^ μS·s^(N)^, however as expected the value of R_ct_ has drastically decreased (99 kΩ).

## Conclusion

The presence of ruthenium oxide, characterized by high redox capacitance and high surface area, improves tested properties of H^+^-selective electrode in contrast to coated-wire electrode. Also, designed solid-contact electrodes present nearly as excellent properties as glass electrode and along with their great performance parameters such as small size and durability, (the electrodes) may be considered to be applied instead of mentioned conventional glass electrode with inner solution.The RuO_2_-based solid-contact pH electrodes show a near Nernstian response to hydrogen ions in the pH range from 2 to 12 with no interferences from monovalent cation (such as Na^+^, K^+^ and Li^+^), which often limit the measuring range of H^+^- selective electrodes.Despite of the fact that the ruthenium oxide is an electronic conductor, examined sensors do not show the redox response.Modified electrodes exhibit remarkable electrical parameters such as low resistance and potential drift along with high capacitance, which results in great potential stability. Obtained parameters’ values unquestionably confirm the beneficial influence of ruthenium oxide on electrodes’ properties.
